# Reliability of anthropometric measurements of a digi‐board in comparison to an analog height board in Namibian children under 5 years

**DOI:** 10.1111/mcn.13677

**Published:** 2024-07-03

**Authors:** Johanna Namene, Christian J. Hunter, Shirley Hodgson, Humphrey Hodgson, Jane Misihairabgwi, Shan Huang, Joel Conkle

**Affiliations:** ^1^ School of Medicine University of Namibia Windhoek Namibia; ^2^ Clinical Care, Education and Research, Centre of Global Health Practice and Impact Georgetown University Washington District of Columbia USA; ^3^ Department of Clinical Genetics St. George's University of London London UK; ^4^ United Kingdom Charity Nutritional Education and Research for Namibia London UK; ^5^ Institute of Liver and Digestive Health, Division of Medicine University College London London UK; ^6^ Maternal, Child and Adolescent Health Program, Burnet Institute Melbourne Victoria Australia; ^7^ Department of Public Health and Preventative Medicine Monash University Melbourne Australia; ^8^ UNICEF Division of Data, Analytics, Planning and Monitoring New York New York USA

**Keywords:** analog, anthropometry, children, digital, malnutrition, measurement, reliability

## Abstract

Poor measurement quality has set back the utility of anthropometry in defining childhood malnutrition, prompting calls for alternative measurement techniques. This study aimed to assess the reliability of anthropometric measurements using a digital height board in comparison to an analog height board in Namibian children under 5 years of age. A cross‐sectional, descriptive study was conducted (*n* = 425) between the age of 6 and 59 months, using anthropometric measurements of weight, height and mid‐upper arm circumference. Two trained enumerators each collected four height measurements of each child: two using an analog height board and two using a digi‐board. The repeated height measurements between and within the enumerators were used to determine intra‐ and interobserver reliability. Reliability of the digi‐board was assessed using the technical error of measurement (TEM), relative TEM (%TEM), intraclass correlation and a Bland–Altman analysis to assess the agreement between the two methods. In all these assessments, the analog height board was considered as the gold standard and used for comparison. The digi‐board showed superiority to the analog height board in terms of reliability (analog TEM = 0.22, digi‐board TEM = 0.16). Although the digi‐board has potential to improve child anthropometry, further clinical and large survey studies are needed to validate the used of this tool in routine population‐based surveys.

## Key messages

- Malnutrition is a global health concern, specifically in children under 5 years, emphasizing the need for interventions to address malnutrition at the population level.
- Moving anthropometric measurement from analog (which is prone to human error) to digital has the potential to improve data quality in anthropometric measurements in children.
- Accurate and reliable measurements are important in identifying populations that are at risk of malnutrition.
- The digi‐board has better reliability compared to the analog height board.
- An increased measurement error was observed in younger children (6–23 months) compared to children between 24 and 59 months of age for both the analog board and the digi‐board.

## Graphical abstract

This study examines the reliability of anthropometric measurements using digital height boards compared to traditional analog boards in Namibian children under five. Results indicate that digital boards show superior reliability, suggesting their potential to improve childhood anthropometric methods; yet, further validation in population surveys is warranted.

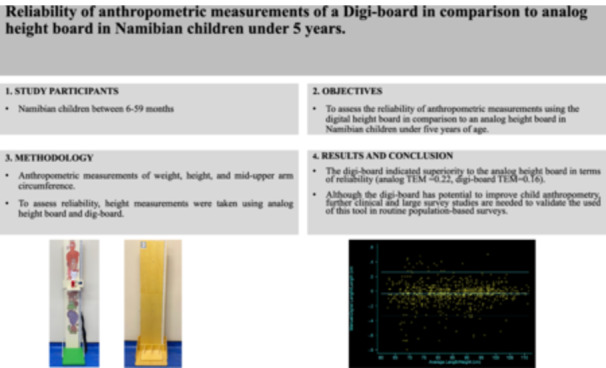

## 1 INTRODUCTION

Malnutrition is a global health concern, particularly in children under 5 years of age (Dewey & Begum, 2011). In research and clinically, child malnutrition is typically identified by taking body measurements and converting those measurements into anthropometric indicators, such as stunting, underweight, overweight and wasting (Park et al., 2009). Determining nutritional status through accurate and reliable anthropometry is of utmost importance to identify high‐risk children who need treatment. Anthropometry is also used to assess the impact of short‐ and long‐term nutrition and health interventions (Gotoa et al., 2015).

Anthropometry currently relies on manual tools, such as wooden height boards, which are prone to human error. This leads to poor quality—resulting in misclassification of individual nutritional status and inaccurate estimates of malnutrition prevalence at the population level (Conkle & Martorell, 2019; Conkle et al., 2017). In addition, data quality varies between countries and between surveys in the same country, making it difficult to meaningfully compare countries, analyse trends over time or target public health interventions (Conkle et al., 2018). In Namibia, the 2015/2016 Namibia Household Income and Expenditure Survey (NHIES) (NSA, 2016) and the 2013 Namibia Demographic Health Survey (NDHS) (MoHSS and ICF International, 2014) both produced excessive biologically implausible measurements of height/length. WHO cut‐offs flagged 12% of children for biologically implausible measurements for the 2013 NDHS and 8.7% for the 2015/2016 NHIES data (Ministry of Health and Social Services, 2019). According to the WHO Expert Committee, surveys with biologically implausible measurements of 1.0% or higher indicate data quality problems (WHO, 1995).

With the usefulness of anthropometry often undermined by poor measurement quality, United Nations International Children's Emergency Fund (UNICEF) called for new technology to improve the measurement of child height. As a result, a prototype digital height board, referred to as the digi‐board, was developed (UNICEF Supply Division, Denmark) for global use. Existing nondigital height boards have limitations, requiring simultaneous attention on reading number lines and child positioning by the measurer (Conkle et al., 2018), which is problematic for infants and young children, who frequently move and resist measurement. Moving height boards to digital, as was already done with weight scales, aims to improve data quality by removing mistakes attributed to reading number lines and allowing measurers to focus more on proper child positioning.

This study evaluated the reliability of digi‐boards compared to the nondigital wooden height boards for children under 5 years of age in Namibia. This study hypothesized that the digi‐board could provide a more reliable way of screening child malnutrition, and we designed the study to contribute to an evidence base that will inform UNICEF on whether or not to replace existing boards with the digi‐boards for surveys and health systems in low‐ and middle‐income countries worldwide.

## 2 MATERIALS AND METHODS

### 2.1 Study design, setting and participants

The recruitment period of this study was from 19 March 2021 until 16 November 2021. Convenience sampling was used, recruiting children under the age of 5 years from mobile clinic centres and kindergartens in and around Windhoek, the capital of Namibia. Informed written consent was obtained from the parents and caregivers of children, and two trained enumerators carried out all data collection procedures.

### 2.2 Sample size

**Figure 1:**
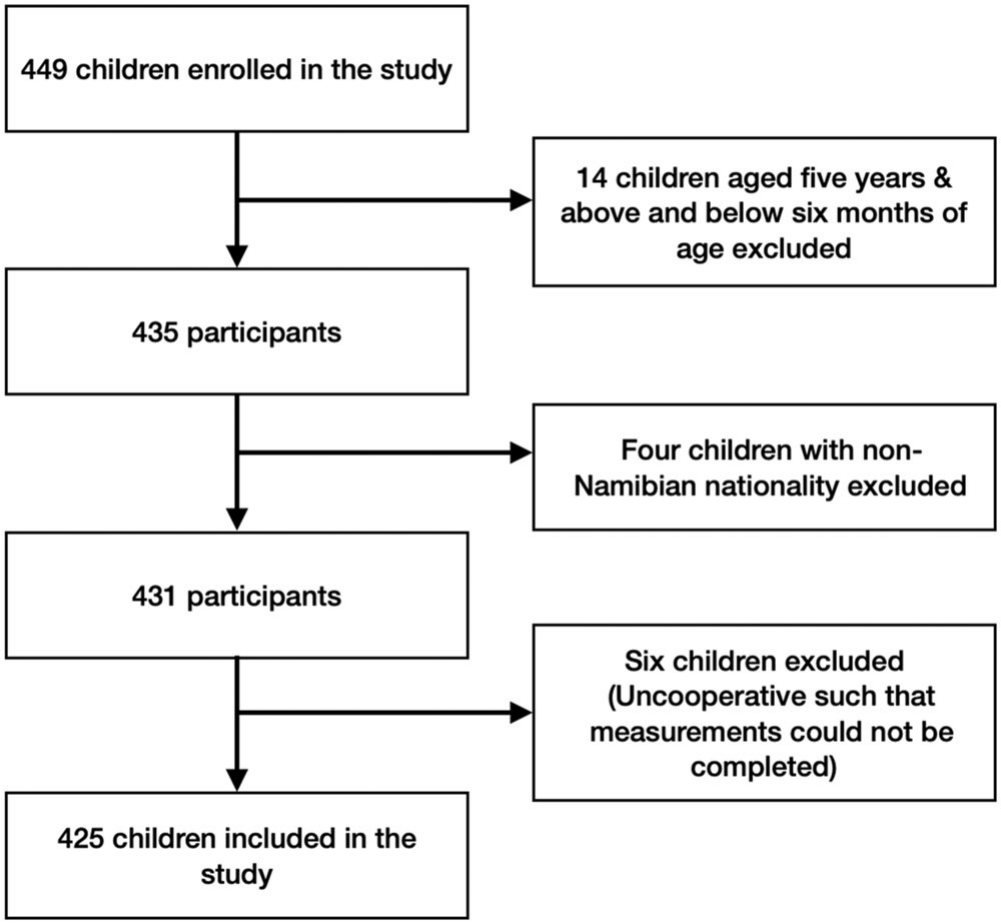
Inclusion and exclusion criteria of the study. Flow chart documenting the inclusion, exclusion criteria and the number of participants included in the study.

The sample size was determined based on information gathered from previous national surveys regarding the nutritional status of Namibian children, according to the Magnani sampling method (Magnani, 1999). The sample size was calculated using the formula *n* = (*t* ^2^ × (1‐*p*))/m^2^ (Magnani, 1999), where *n* is the required sample size, *t* is the confidence level at 95% (standard value of 1.96), *p* is the estimated prevalence of malnutrition in the project area and m is the margin of error at 5% (standard value of 0.05), yielding a total of 369 participants. However, a total of 425 participants were recruited successfully into this study. Despite reaching the intended sample size, there was a high level of interest from parents to have their children measured. Given the availability of sufficient resources, the decision was made to continue data collection beyond the initial sample size. Figure 1 shows the exclusion and inclusion criteria for the study.

### 2.3 Data collection procedure: Anthropometry

The investigator provided detailed information on the study to the children's parent/caregiver and those who agreed for their children to participate in the study signed the consent form. Attached to the informed consent was the data collection sheet, where the parent/caregiver was asked to provide the demographic information of the child, including the date of data collection, name, date of birth, age, sex and the parent/caregiver's names and contact information. Parents were asked to provide children's health information passport to confirm the date of birth. The contact information of the parents/caregivers was requested to enable communication with them in cases where the child was malnourished, facilitating immediate medical attention. With the help of the parent/caregiver, children were prepared for measurements, by removing heavy clothing, shoes and untying their hair. The children's MUAC was then taken, followed by weight measurement and then height/length. All the measurements were entered manually into the data collection sheet. To facilitate accurate data entry, each participant was given a quick response (QR) code, which was printed on their data collection sheet and informed consent. After fieldwork, data were captured in a Microsoft Excel sheet for analysis.

**Figure 2:**
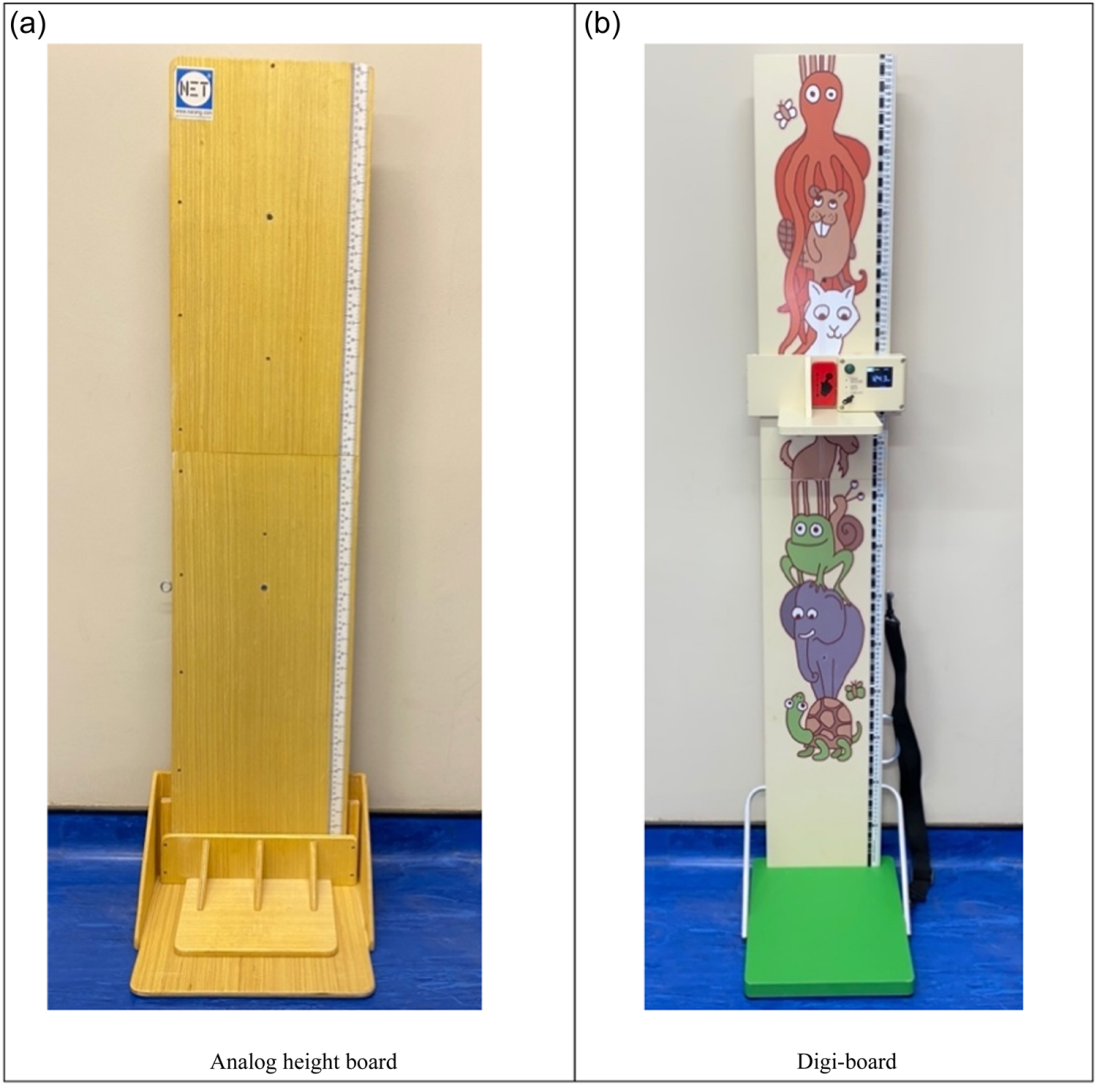
Anthropometric height boards. (a) is an analog (non‐digital) height board that was used as the WHO gold standard. (b) is a new digital height board (named digi‐board). Both instruments were used to measure the length and height of children under 5 years of age.

Weight, height/length and mid upper arm circumference (MUAC) were measured for each participant according to the WHO standards (WHO, 1995). Weight was measured using a portable digital scale (ADE) to the nearest 0.1 kg. MUAC was measured using a MUAC tape (UNICEF Supply Division) by wrapping tape around the participant's arm at the mid‐point of the upper arm, between the acromion process and the tip of the olecranon process, to the nearest 0.1 cm. Standing height was measured for children above 2 years of age, while recumbent length was measured for children below 2 years of age to the nearest 0.1 cm. An analog height board (Figure 2a) (UNICEF Supply Division, Copenhagen, Denmark) and a new digital height board (PKP Bardejov s.r.o, Slovakia and UNICEF Supply Division), hereafter referred to as the digi‐board (Figure 2b), were also used to collect height measurements on each participant. The height measurements from the analog height board (WHO gold standard) were used to compare measurements from the digi‐board in terms of reliability.

Before commencing fieldwork, two enumerators underwent training in the proper use of all manual equipment (analog height board, digi‐board). This training involved viewing of instructional videos from the WHO anthropometry training course and Centers for Disease Control and Prevention (CDC) on anthropometric measurements. Subsequently, they received hands‐on training from an expert anthropometrist, who is a child nutrition specialist affiliated with UNICEF. During this training, both enumerators were educated on the principles of growth monitoring and the importance of obtaining parental consent before involving children in the project.

Length measurements of children were carried out as follows: The height board was placed on a hard surface such as the ground, floor or a solid table, ensuring that the measuring board was stable. Enumerators placed the questionnaire and pen/pencil within easy access for recording the measurement. Both enumerators gently lowered the child onto the measuring board, providing support to the child's body and the head. Before the measurement was taken, the enumerators ensured that the positioning of the child on the board was correct: (a) the head of the child was placed against the base of the boards, while the feet were placed flat against the foot piece; (b) it was ensured that the child looked straight up, such that the line of sight was perpendicular to the board; (c) the child was placed at the centre of the board, with the legs straight together and the knees and the feet together; and (d) one enumerator held the child's head in position, while the other gently held the child's legs by the knees with one hand to keep them straight as the other hand moved the foot piece to take the measurements. Once the enumerator was satisfied that the child's position was correct, the measurement was recorded on the questionnaire.

In order to assess the reliability of the two height boards, each enumerator took four measurements of each child. Two consecutive measurements were taken using the analog height board and two consecutive measurements were taken using the digi‐board. The child was positioned on the height board for the first measurement, using both the analog and the digi‐board. Then, children took a break while the next child was positioned for their first measurement. After the break, the children were called back and repositioned on the height boards for the second measurement to be taken. This was done to test the reliability of measurements within (intra‐observer) and between (interobserver) measurers for both types of height boards.

### 2.4 Statistical analysis

Data were imported into both SPSS version 27 (IBM Corp.) and GraphPad Prism 9.3.1 (GraphPad Software Inc) for quantitative analysis. To determine the children's nutritional status, measurements of height, weight and MUAC were entered into ENA software (SMART Methodology) and the anthropometric z‐scores of each child were derived. The z‐scores provided an indication of the nutritional status of a child according to 2006 WHO growth reference standards (WHO, 2006). To measure reliability, the technical error of measurement (TEM), the relative TEM (%TEM) and the intraclass correlation (ICC) were calculated using SPSS version 27. In order to calculate TEM, at least two measurements of the same child should be taken by the same observer (intraobserver reliability) or by at least two observers taking the same measurement on the same child (interobserver reliability).

Calculations of TEM for both intra‐ and interobserver reliability are the same when only two observers are involved or when two measurements are taken. The equation is as follows: $\mathrm{TEM}=\sqrt{(D^{2})/2N}$, where *D* is the difference between measurements and *N* is the number of participants measured (Stomfai et al., 2011; Ulijaszek & Kerr, 1999). TEM for interobserver reliability was calculated by determining the differences between the averages of the 1st measurement of enumerator 1 and the 1st measurement of enumerator 2 and between the 2nd measurement of enumerator 1 and the 2nd measurement of enumerator 2. TEM analyses the standard deviation between repeated measurements obtained from the two methods (analog and digi‐board) on the same child. In order to compare TEM collected from different measurements, absolute TEM was converted into relative TEM (%TEM) using the following equation: %TEM = (TEM/mean) × 100 (Stomfai et al., 2011; Ulijaszek & Kerr, 1999). ICC is another measure of reliability, with values ranging from 0 to 1, where values close to 1 indicate little error (Ulijaszek & Kerr, 1999). ICC was obtained using Intraclass correlation analysis on SPSS. To test whether the difference in reliability between digital and analog boards was statistically significant, we calculated the mean absolute difference of two measurements for each type of board and then used the Wilcoxon statistical test (Cleophas & Zwinderman, 2016) in SPSS to obtain a p‐value comparing the absolute differences for the two types of boards. A *p*‐value of <0.05 was considered significant for the differences between the two height boards.

## 3 RESULTS

### 3.1 Characteristics of children under 5 years

A total of 425 children were recruited into the study; however, 15 children did not have two measurements and hence were excluded from the reliability analysis. Therefore, the final sample size consists of 410 children who were measured by two different enumerators, providing two observations each, hence 820 measurements in total. Sample characteristics and the prevalence of malnutrition in the study are presented in Table 1. Children (*n* = 425) were divided into two groups according to their ages, that is, under 2 years of age (6–23 months) and over 2 years of age (24–59 months).

**Table 1:** Sample characteristics of children under 5 years of age.

Age in months	*N*	Mean (range)
All	425	28.9 (6.0–59.0)
Age group (months)	*n*	%
6–23.9	174	40.9
24–59.9	251	59.1
Sex	*n*	%
Female	214	50.4
Male	211	49.6
Ethno linguistic group	*n*	%
Bushman, Damara & Nama	47	11.1
Herero	26	6.1
Nyemba & Rukwangari	4	0.9
Mixed	83	19.3
Owambo	265	62.4

Nutritional status, *n* = 425, no (%)	
Wasting (<−2 SD WHZ)	18 (4.2)
Underweight (<−2 SD WAZ)	70 (16.5)
Overweight (<−2 SD WHZ)	10 (2.4)
Stunting (>2 SD HAZ)	126 (29.6)

Abbreviations: HAZ, height for age; WAZ, weight for age; WHZ, weight for height.

Of all the 425 children recruited in this study, 40.9% were under the age of 2 years and 59.1% were above 2 years of age. There was a low prevalence of wasting (4.2%) and overweight (2.4%), a medium prevalence of underweight (16.5%) and a high prevalence of stunting (29.6%) (De Onis et al., 2019).

### 3.2 Reliability

Table 2 presents both intra‐ and interobserver reliability for length and height measurements, disaggregated by age groups. Reliability is known to be good when the variability between repeated measurements of the same subject by one (intraobserver differences) or two or more (interobserver differences) observers is low (Stomfai et al., 2011).

**Table 2:** Intra‐ and interobserver reliability for length or height: Mean, standard deviation, mean absolute difference, intratechnical error measurement (intra‐TEM), intertechnical error measurement (inter‐TEM), relative TEM and intraclass correlation coefficient (ICC) for children 6–59 months of age using analog and digital height boards.

Age group (months)		Mean (SD) (cm)^b^		Mean (SD) absolute difference (cm)^c^			Technical error of measurement (TEM) (cm)^d^				Relative TEM (%TEM)^e^		Intraclass correlation coefficient (ICC) (95% CI)^f^	
Stature (length or height)	No. of measurements^a^	Analog	Digital	Analog	Digital	*p*‐Value	Analog	95% precision margin	Digital	95% precision margin	Analog	Digital	Analog	Digital
Intraobserver reliability of stature (length or height)														
All (6–59.9 months)	820	85.0 (11.6)	85.0 (11.6)	0.20 (0.24)	0.15 (0.17)	<0.001	0.22	0.43	0.16	0.31	0.25	0.18	0.999 (0.999–0.999)	1 (1.000–1.000)
6–23 months (length)	322	73.5 (5.8)	73.5 (5.8)	0.29 (0.30)	0.20 (0.23)	<0.001	0.29	0.57	0.21	0.41	0.39	0.29	0.997 (0.997–0.998)	0.999 (0.998–0.999)
24–59.9 months (height)	498	92.5 (7.6)	92.5 (7.7)	0.15 (0.17)	0.11 (0.12)	<0.001	0.15	0.29	0.11	0.22	0.16	0.12	0.998 (0.998–0.998)	1 (1.000–1.000)
Interobserver reliability of stature (length or height)														
All (6–59.9 months)	820	85.0 (11.6)	85.0 (11.6)	0.19 (0.15)	0.15 (0.20)	<0.001	0.22	0.43	0.17	0.33	0.26	0.20	1 (1.000–1.000)	1 (1.000–1.000)
6–23 months (length)	322	73.5 (5.8)	73.5 (5.8)	0.26 (0.33)	0.18 (0.23)	<0.001	0.30	0.59	0.19	0.37	0.40	0.27	0.997 (0.997–0.998)	0.999 (0.999–0.999)
24–59.9 months (height)	498	92.5 (7.6)	92.5 (7.7)	0.15 (0.17)	0.13 (0.18)	0.004	0.16	0.31	0.16	0.31	0.18	0.17	1 (0.999–1.000)	1 (0.999–1.000)

^a^ No. of measurements: the initial sample size is 425 children, but 15 children did not have two measurements and hence were excluded from this analysis. Therefore, the final sample size is 410 children who were measured by two different enumerators, providing two observations each, thus a sample size of 820 children in total.

^b^ Mean (SD): mean of two analog and two digital measurements (enumerator 1 first and second measurements and enumerator 2 first and second measurements).

^c^ Mean absolute difference: mean of the absolute difference between measurement 1 and measurement 2.

^d^ TEM: Square root of measurement error variance, $\mathrm{TEM}=\sqrt{(D^{2})/2N}$, where *D* is the difference between measurements and *N* is the number of individuals measured.

^e^ % TEM = (TEM/mean) × 100.

^f^ ICC (95% confidence interval): Variability between measurement 1 and measurement 2, used the ICC single measure with an absolute agreement definition.

In comparison to the analog board intra‐observer TEM (0.22), the digi‐board TEM (0.16) was lower, indicating that the digi‐board showed better reliability for repeated measurements by the same enumerator. The 95% precision margin showed that when using a digi‐board, the enumerator's second measurement was within ±0.31 cm of their first measurement 95% of the time for the entire sampled population compared to ±0.43 cm for the analog board. Comparison of the intraobserver mean absolute differences showed that the smaller difference between two measurements for the digi‐board (0.15 cm) compared to the analog board (0.20 cm) was statistically significant (*p* < 0.001).

For interobserver reliability, the digi‐board TEM (0.17) was lower than the analog board TEM (0.22), again indicating that the digi‐board showed better reliability in comparison to the analog board. When using a digi‐board, one enumerator's measurement was within ±0.33 cm of the other enumerator's measurement 95% of the time, compared to ±0.43 cm when using an analog board. Similar to intra‐observer reliability, comparison of the interobserver mean absolute differences showed that the smaller difference between two measurements for the digi‐board (0.15 cm) compared to the analog board (0.19 cm) was statistically significant (*p* < 0.001).

While the intra‐ and interobserver relative TEM values of the 6–23 months age group were lower when using the digi‐board compared to the analog board, the relative TEM for interobserver reliability was nearly similar for both the analog (0.18%) and digi‐board for the 24–59 months age group (0.17%). Differences in reliability in the two age groups were observed, with worse inter‐ and intraobserver reliability in the younger age group compared to the older age group for both types of boards. For intraobserver reliability, the relative TEM (0.39%) was higher in the 6–23 months age group in comparison to the 24–59 months age group (0.16%) for the analog boards. For the digi‐boards, the relative TEM was also higher in the 6–23 months age group (0.29%) compared to the 24–59 months age group (0.12%). This indicates that reliability was better in the 24–59 months age group compared to the 6–23 months age group when using both types of height boards.

Similar to intraobserver reliability, the interobserver relative TEM was higher in the 6–23 months age group (0.40%) compared to the 24–59 months age group (0.18%) for the analog boards and 0.27% for the 6–23 months age group and 0.17% for 24–59 months age group for the digi‐board. Better intra‐ and interobserver reliability in the older age group was expected because older children tend to be more compliant during measurements.

### 3.3 Bias—agreement between digital and analog boards

The Bland–Altman comparison of the analog and digital height measurements is plotted in Figure 2. This was done to measure the agreement between the measurements of the two methods. The overall mean difference in the two methods was –0.036 cm (95% confidence interval [CI] −0.047; −0.026). This is an indication that on average, the analog height board yielded slightly shorter measurements for children, but the difference was too small to be considered meaningful. The 95% limit of agreement was between −0.334 and 0.261 cm. This means that the difference in measurements between the analog and digital height boards falls between −0.334 and 0.261 cm 95% of the time and that 95% of future differences between measurements of the same participants will fall within these limits. Pitman's Test of Difference in variance yielded a *p*‐value of 0.075, indicating high likelihood that the difference between analog and digital measurements was dependent on children's height. Visually, the graph shows more variation among shorter children (who are also younger) compared to taller children, indicating less agreement among younger children. This finding aligns with the result of poorer reliability of anthropometric measurements among younger children.

**Figure 3:**
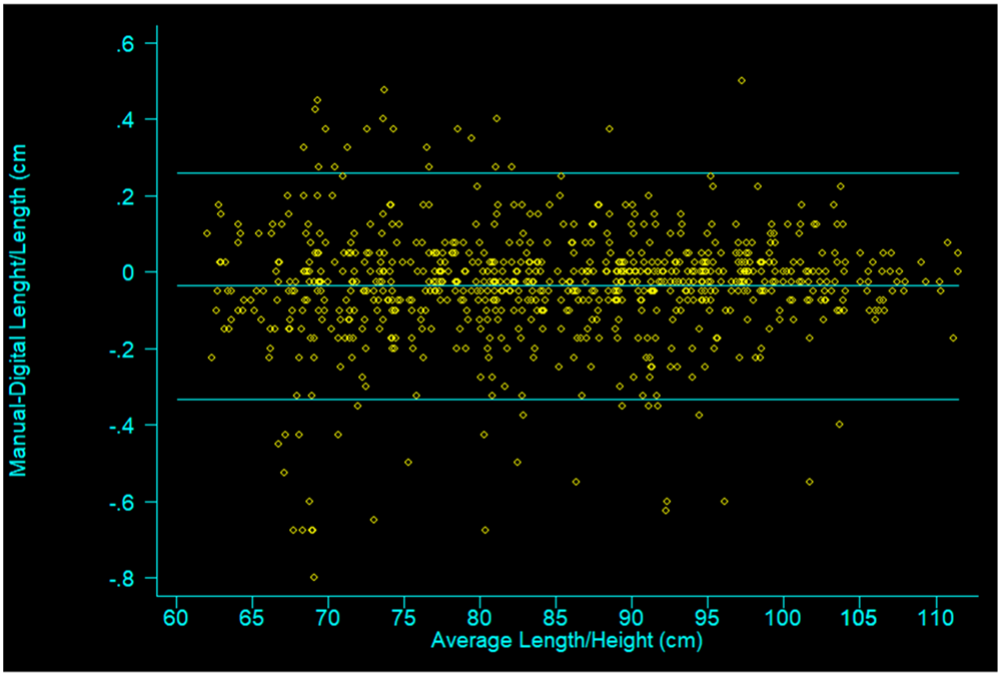
Bland–Altman plot. Analog and digital height/length measurements (*y*‐axis) plotted against average measurements (*x*‐axis) of children under 5 years of age, *n* = 410.

## 4 DISCUSSION

The findings of this study indicate that both the analog height board and the digi‐board demonstrate low levels of measurement error and a high level of reliability, as indicated by their %TEM measurements, both of which fall within the acceptable level of reliability, defined as %TEM < 2% (Jamaiyah et al., 2010; Perini et al., 2005). While reliability is high for both height boards, the digi‐board demonstrated better reliability compared to the analog height board. These findings suggest that the digi‐board has the potential to reduce human errors that can occur when using the analog height board.

The digi‐board is a digital innovation meant to improve the quality of child anthropometry. Our results suggest that the digi‐board has the potential to improve anthropometric measurements, which is crucial in healthcare settings and research, where precise measurements are necessary for monitoring growth or assessing health conditions (Viviani et al., 2018), and large national surveys, where reliability measurements are essential for accurately identifying levels of malnutrition in the country.

### 4.1 Secondary findings and variations in younger age groups

Our study reported an agreement of measurements between the digi‐board and the analog height board, but with Bland–Altman statistics indicating less agreement in younger children. Unsurprisingly, reliability was also poorer in the younger age group. Both intra‐ and in interobserver relative TEM values were higher in children 6–23 months of age than in children between 24 and 59 months of age for both types of boards.

The observed discrepancy can potentially be attributed to the fact that older children tend to be more compliant during measurements. Younger children may struggle to remain still during measurements, frequently cry and cannot follow instructions. This makes it difficult for the enumerator to focus on both positioning the child and concentrating on taking the reading. We did not find meaningful systematic bias (e.g., analog boards consistently overestimated the height/lenght measurements of children, making them appear much taller than their actual height). Rather, we found higher random variability in measurements among younger children, and evidence that the issue was also related to the board itself, as the analog height board was less reliable when measuring younger children compared to the digi‐board. We attributed the lower reliability to younger children kicking, which may have impacted the analog height board more than the digi‐board because of the board itself (the head/foot piece was not firm/fixed enough on the analog board) and because with the analog board, the measurer has to take a couple of extra seconds to look away from the head/foot piece to read the measurement, whereas with the digi‐board, the measurer can push a button to lock in the measurement while maintaining focus on the head/foot piece position.

Our findings are in agreement with similar studies demonstrating increased measurement error in younger children (Carsley et al., 2019; Rios‐Leyvraz et al., 2022) and align with studies focusing on the reliability of anthropometric measurements in children, suggesting that younger children have higher levels of measurement errors because they are unable to co‐operate with measurements compared to older children (Bougma et al., 2022; Gupta et al., 2020; Walker et al., 2013).

### 4.2 Enumerator observations

According to the enumerators' field experience, it was quicker and easier to measure children when using the digi‐board compared to the analog board, because once the child was positioned and ready for measurement, all the enumerators had to do was click one button and the measurement was displayed on the screen. In contrast, with the analog height scale, the enumerator needed time to adjust their eyes to the measuring scale to manually take the reading. This may have contributed to better reliability of the digi‐board compared to the analog height board. Another factor that could have affected reliability was that children were attracted to animal drawings that were only on the digi‐board (Figure 1), with some children wanting to play with the images when placed on the board. This impacted the children's behaviour and increased their comfort during measurements, making them calmer and the measurement easier (Figure 3).

### 4.3 Strengths and limitations of the study

To our knowledge, this study is the first to compare digital and analog height boards in a low‐ or middle‐income country setting. As such, the study is a starting point to build the evidence base on digital height boards in those settings and can be used to inform future studies using similar technology. Our study had sufficient sample size (425 children) for disaggregated analysis by age group, and the design allowed for assessment of accuracy and both intra‐ and interobserver reliability.

It is worth noting that the study did not include children under 6 months of age. Therefore, the findings regarding the digi‐board's reliability may not be generalized to all children under the age of 5 years. Another limitation was the fact that this study only included two enumerators, exclusive to the study, which was a strength with respect to ensuring adherence to the study protocol but also a limitation in terms of the generalizability of findings. Other enumerators could have different experiences with use of the equipment. In addition, since the study was not carried out by staff in a clinical or survey setting, we cannot extrapolate the findings to the specific contexts where the equipment is meant to be adopted.

### 4.4 Unanswered questions and potential future research

Further testing of the digi‐board in real‐world contexts is needed to validate its potential benefits fully. The next step is to use the digi‐board in clinical and survey settings to assess accuracy, reliability and practicality when used outside of a research setting.

Our study did not investigate the time required to complete measurements between the two height boards, nor did we assess costs. The issues of efficiency and affordability will certainly affect whether digital height boards are widely adopted, and future research can assess costs and quantify potential gains in terms of efficiency observed by the enumerators in this study. Future research could also explore the impact of children's behaviour during measurements on reliability and the experiences and perceptions of healthcare workers, caregivers and children when using the digi‐board compared to the analog height board to better inform implementation strategies.

## 5 CONCLUSION

A current, standard tool for child anthropometry, the analog height board, can introduce measurement errors due to reliance on manual readings, which can lead to misreading and/or improper positioning of the child. Our study showed that a digital height board has the potential to improve the quality of anthropometric measurements of children but it needs to be studied further in real‐world, clinical and survey settings. UNICEF is currently studying the use of the digi‐board in a large‐scale survey in Eswatini. Additional research on the use of digital height boards in surveys and clinics in other settings is needed to estimate the impact of using a digital height board on the quality of anthropometric measurements, which can inform widespread adoption of the innovation.

Although the enumerators preferred the digi‐board for measurement, some negative findings were observed and noted. Measurements with the digi‐board were impacted as the head piece was observed to frequently detach from the instrument's vertical board. Although detachment could be avoided by carefully moving the head piece, this flaw should be fixed before recommending widespread use of the new board. In addition, it is important to highlight that the digi board requires power supply for it to function and for this reason, this may limit its use in situations where the device is not adequately charged or when access to electricity is limited, especially in remote areas. However, once it is fully charged, the battery can last approximately 4–5 days with continuous full‐day use, mitigating concerns regarding immediate power availability.

## AUTHOR CONTRIBUTIONS

Johanna Namene, Joel Conkle, Christian J. Hunter, Shirley Hodgson and Humphrey Hodgson were involved in the conceptualization and design of the research study and protocol. Johanna Namene performed the data collection and project administration. Johanna Namene and Joel Conkle wrote the original draft of the paper. Johanna Namene, Joel Conkle, Christian J. Hunter, Shirley Hodgson, Humphrey Hodgson, Shan Huang and Jane Misihairabgwi reviewed and edited the manuscript. Johanna Namene and Joel Conkle performed data analysis. Joel Conkle, Christian J. Hunter, Jane Misihairabgwi, Shirley Hodgson and Humphrey Hodgson supervised the research study.

## CONFLICT OF INTEREST STATEMENT

The authors declare no conflict of interest.

## ETHICS STATEMENT

This study was conducted in accordance with the Declaration of Helsinki and was approved by the Institutional Review Board (or Ethics Committee) of the University of Namibia (protocol code H‐G/581/2020, on 31 August 2020) and the Ministry of Health and Social Services (protocol code 17/3/3/JN, on 03 December 2020).

## Data Availability

The data that support the findings of this study are available from the corresponding author upon reasonable request.
